# Effect of a web-based audit and feedback intervention with outreach visits on the clinical performance of multidisciplinary teams: a cluster-randomized trial in cardiac rehabilitation

**DOI:** 10.1186/s13012-016-0516-1

**Published:** 2016-12-09

**Authors:** Wouter T. Gude, Mariëtte M. van Engen-Verheul, Sabine N. van der Veer, Hareld M. C. Kemps, Monique W. M. Jaspers, Nicolette F. de Keizer, Niels Peek

**Affiliations:** 1 Department of Medical Informatics, Academic Medical Center/University of Amsterdam, Room J1B-127. Meibergdreef 9, 1105 AZ Amsterdam, The Netherlands; 2 MRC Health eResearch Centre, Division of Informatics, Imaging and Data Sciences, The University of Manchester, Manchester Academic Health Science Centre, Manchester, UK; 3 Department of Cardiology, Máxima Medical Centre, Veldhoven, The Netherlands

**Keywords:** Quality improvement, Quality indicators, Health care, Cardiac rehabilitation, Guideline adherence, Feedback

## Abstract

**Background:**

The objective of this study was to assess the effect of a web-based audit and feedback (A&F) intervention with outreach visits to support decision-making by multidisciplinary teams.

**Methods:**

We performed a multicentre cluster-randomized trial within the field of comprehensive cardiac rehabilitation (CR) in the Netherlands. Our participants were multidisciplinary teams in Dutch CR centres who were enrolled in the study between July 2012 and December 2013 and received the intervention for at least 1 year. The intervention included web-based A&F with feedback on clinical performance, facilities for goal setting and action planning, and educational outreach visits. Teams were randomized either to receive feedback that was limited to psychosocial rehabilitation (study group A) or to physical rehabilitation (study group B). The main outcome measure was the difference in performance between study groups in 11 care processes and six patient outcomes, measured at patient level. Secondary outcomes included effects on guideline concordance for the four main CR therapies.

**Results:**

Data from 18 centres (14,847 patients) were analysed, of which 12 centres (9353 patients) were assigned to group A and six (5494 patients) to group B. During the intervention, a total of 233 quality improvement goals was identified by participating teams, of which 49 (21%) were achieved during the study period. Except for a modest improvement in data completeness (4.5% improvement per year; 95% CI 0.65 to 8.36), we found no effect of our intervention on any of our primary or secondary outcome measures.

**Conclusions:**

Within a multidisciplinary setting, our web-based A&F intervention engaged teams to define local performance improvement goals but failed to support them in actually completing the improvement actions that were needed to achieve those goals. Future research should focus on improving the actionability of feedback on clinical performance and on addressing the socio-technical perspective of the implementation process.

**Trial registration:**

NTR3251

## Background

The number of chronically ill patients is increasing, requiring hospitals to reconsider their role and responsibility in chronic disease management [1, 2]. At the same time, health organizations are under public pressure to increase their accountability and to deliver optimally efficient and effective care [3]. The field of cardiac rehabilitation (CR) typically faces these challenges. CR offers cardiovascular disease patients a need-based, cost-effective, multidisciplinary approach to regain physical capacity, improve psychosocial condition, achieve lifestyle changes, and reduce future cardiovascular risk [4–7]. The efficacy of CR has been studied extensively [6, 8] and was recently shown to be associated with a substantial survival benefit [9]. However, lack of guideline concordance limits the ability of CR to reach its full potential [10–12]; computerized clinical decision support (CDS) has previously been shown to have the potential to improve this [13]. However, considerable non-concordance remained due to organizational and procedural barriers not being addressed because individual CDS users considered them beyond their own influence and responsibility [14]. This finding stressed the need for an intervention specifically directed at decision-making processes at the team rather than at an individual level. This coincides with the approach advised by the American Heart Association (AHA) [11], advocating that entire multidisciplinary CR teams should implement coordinated, joint efforts to reinforce the importance of outpatient CR among healthcare systems, providers, and the public [11].

The AHA also promotes the use of quality indicators to monitor and improve clinical performance, for example using audit and feedback (A&F) strategies. A&F involves providing professionals with periodic objective summaries of their clinical performance [15] and is considered to be effective because it can support professionals in assessing their own clinical performance [15]. Previous studies suggested A&F to be the most effective if feedback is provided by a supervisor or colleague, more than once, both verbally and in writing; if baseline performance is low; if it includes explicit goals and an action plan; and if combined with educational meetings [15–18]. Other suggested effect modifiers are the perceived quality of the data underlying the feedback, motivation, and interest of the recipient, organizational support for quality improvement (QI), and the way in which performance targets or benchmarks are derived [19].

We used these successful characteristics described in the literature [15–19] to guide the development of a multifaceted A&F intervention to improve clinical performance in the field of CR in the Netherlands [20]. To further maximize its effect, our intervention specifically focused on engaging multidisciplinary teams and their managers rather than individual professionals [20]. The objective of this study was to assess the effectiveness of the multifaceted A&F intervention in a cluster-randomized trial among CR centres in the Netherlands. We measured effects on 11 care processes and six patient outcomes for CR (primary outcomes). Our secondary outcomes included overall performance, data completeness, and difference in guideline concordance with respect to prescribing CR therapies.

## Methods

### Study design

Centres participating in the trial were randomized to receive feedback limited to either psychosocial rehabilitation (disease-specific education and lifestyle modification; study group A) or physical rehabilitation (exercise training and relaxation and stress management training; study group B). In this way, both groups received an intervention, whilst serving as each other’s control. We refer to the study protocol for further details of the experimental design [20].

### Eligibility of participants

Dutch CR centres working with an electronic patient record (EPR) system for CR were eligible to participate. Multidisciplinary CR teams included cardiologists, physical therapists, nurses, psychologists, dieticians, social workers, and/or rehabilitation physicians. Teams were required to allocate dedicated time for study activities from at least the local CR coordinator (usually a specialized nurse), a cardiologist, one professional from another discipline, and the centre’s manager. Recruitment took place from July 2012 until December 2013. All CR patients who started rehabilitation in one of the participating centres during the study period were eligible for inclusion in our analyses. CR is recommended for all patients who have been hospitalized for an acute coronary syndrome (ACS) and for those who have undergone a cardiac intervention [5, 21]. Patients entering outpatient CR in the Netherlands are offered a comprehensive, individualized rehabilitation programme with a typical duration of 6–12 weeks, consisting of one or more of the four group-based therapies supplemented by individual counselling when indicated. Consistent with international guidelines, the Dutch guidelines for CR [22, 23] state that the individualized programme should be based on a need assessment procedure where data items concerning the patient’s physical and psychosocial condition are gathered.

### Intervention

Our intervention comprised three main components: (i) periodic performance feedback reports, (ii) goal setting and action planning, and (iii) educational outreach visits. To facilitate the first two components, we developed a web-based system called ‘CARDSS Online’ [24]. Participants were requested to upload their anonymized patient data quarterly, after which the system created new feedback reports. Within days after new reports were released, educational outreach visits were held with the local multidisciplinary team to reflect on the feedback, to set goals and plan actions, and to update existing action plans following a continuous A&F improvement cycle [25]. Participants were offered four iterations of this cycle facilitated by a researcher through outreach visits, as well as up to two additional iterations facilitated via telephone.

#### Feedback reports

**Fig. 1:**
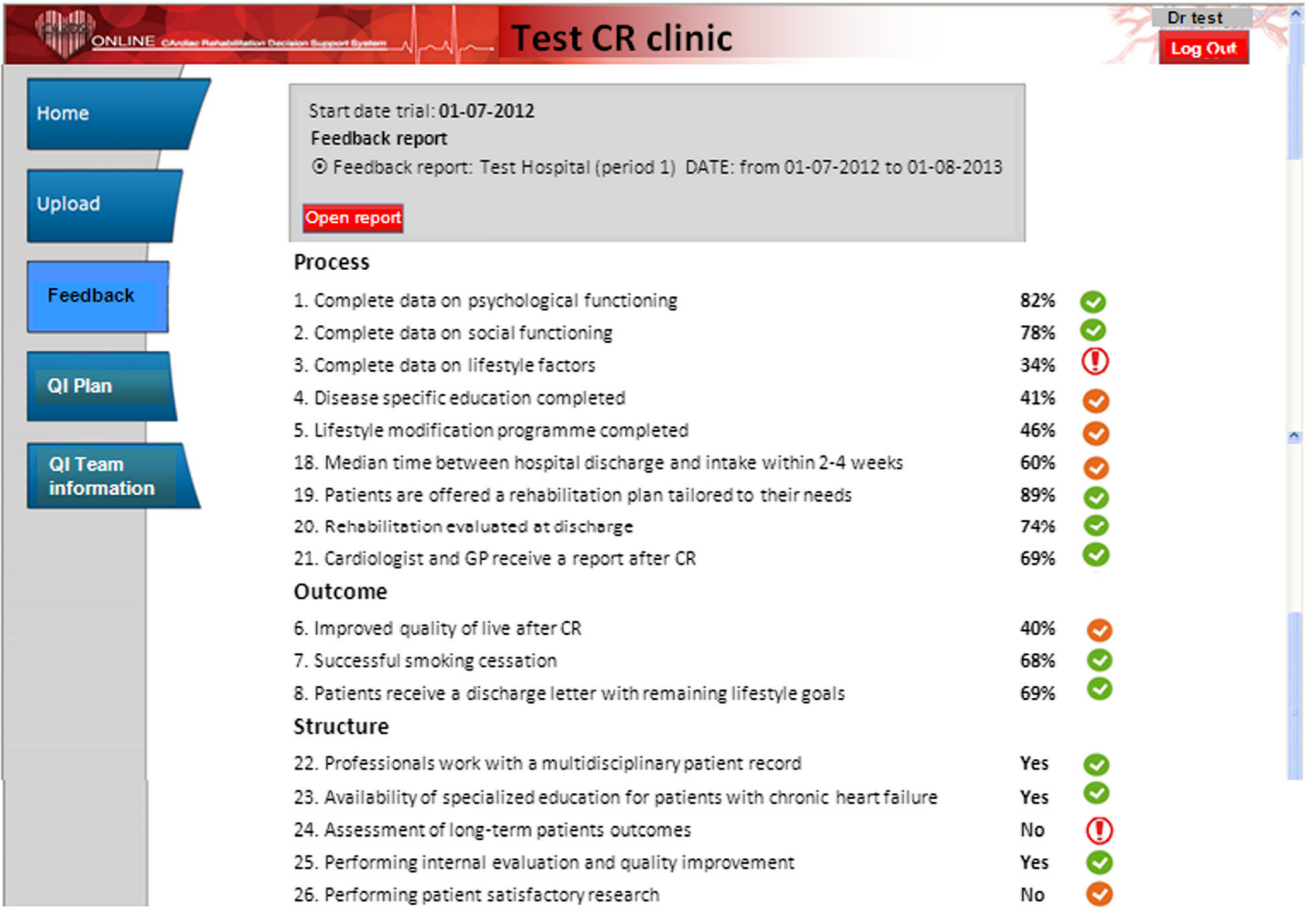
Example feedback report for a centre in study group A. Group A received feedback on performance in the psychosocial rehabilitation (indicators 1 through 8) and on general processes (indicators 18 through 21) and structures (indicators 22 through 26). The indicator scores in this report are fictitious but representative for the scores seen in real reports. *Abbreviations*: *CR* cardiac rehabilitation, *GP* general practitioner

Feedback reports available through CARDSS Online consisted of performance scores on a set of indicators; each indicator represented a care process or patient outcome for CR. The performance scores were accompanied by benchmark information represented by ‘traffic light’ coloured icons (Fig. 1). Red, yellow, or green colours were assigned based on the centre’s performance score relative to peer performance using the concept of achievable benchmarks [20]. A grey colour was assigned if there were insufficient data (<10 patients) available to compute a score. The processes and outcomes in the indicator set were defined in close collaboration with a panel of CR professionals [26]. The eight indicators related to psychosocial rehabilitation were only shown to centres in group A, whereas group B only saw the nine indicators related to physical rehabilitation (Appendix 1). All these processes and outcomes were measured as dichotomous variables at patient level. We also fed back nine indicators related to general CR processes (four patients and five centre levels) to centres in both groups (see Appendix 2).

#### Goal setting and action planning

After receiving a feedback report, participants could use CARDSS Online to develop a QI plan by selecting indicator areas for improvement (related to quality indicators in the feedback report). For each area they targeted, they could specify the problem and its presumed causes, set an improvement goal, and plan concrete actions on how to reach that goal. Actions were assigned to specific team members and were set with a due date. At each A&F iteration, the QI plan was updated by marking actions as ‘completed’, ‘cancelled’, or ‘in progress’, by planning new actions, or by adding new areas for improvement to the plan. If all actions for a specific improvement area were completed, that area was removed from the QI plan.

#### Educational outreach visits

Educational outreach visits were conducted by one investigator (MvE or WG; both with a non-clinical, QI background) and typically lasted 2.5 h. All members of the local multidisciplinary team were invited to attend this session, and the visits always had the same structure. First, the investigator gave a short presentation to explain the purpose of the visit and the intervention. Next, the team discussed and reflected upon their most recent feedback report and created or updated their QI plan. The role of the investigator was to answer questions about the feedback (e.g. patient inclusion or exclusion criteria for specific indicators), help teams to plan actions that were achievable within the study period, and upon request provide lists of patients who had not received the recommended clinical practice or experienced outcome of interest for a specific indicator.

### Outcome measures

Our primary outcome was the difference in improvement between the two study groups with respect to each of the 17 indicators (11 care processes and six patient outcomes) for which exactly one study group received feedback. First, we evaluated improvement per indicator at patient level; additionally, we compared, at centre level, overall performance (number of indicators at or above benchmark level) and data completeness (number of indicators for which centres recorded complete data) at baseline and 1 year of follow-up.

Secondary outcome measure was the difference in change in guideline concordance with respect to prescribing the four main CR therapies. Concordant prescribing was defined as prescribing a therapy for patients who were indicated to receive it and not prescribing a therapy for patients who were not indicated to receive it according to the Dutch clinical CR guidelines [22, 23]. Additionally, we measured change in concordance with respect to actual attendance of these four therapies by patients.

### Patient involvement

To ensure that patients’ perspectives were reflected in the intervention, patients were involved in the development of the quality indicators that were used to give feedback to CR professionals and also served as primary outcome measures of the study [26].

### Data collection and validation

We used routinely collected patient data from centres’ EPRs. At the time we conducted our study, two commercial vendors of EPR systems for CR were available in the Netherlands. Both systems incorporated the Dutch CR guidelines [22, 23] and followed the same data model. Data collection was structured as part of the needs assessment procedure and fed into the CDS module providing prescription recommendations for each of the four CR therapies [27].

Centres participated for a minimum of 1 year, with data collection ending in December 2014. At the end of the trial, we performed an audit to assess data quality and completeness by comparing our study database to an independent data source (typically the centres’ local patient clinic schedules). From the analyses for each of the four CR therapies, we omitted centres with more than 25% discrepancies between the study database and the independent data source for prescribing that therapy. For further details, we refer to the study protocol [20].

### Sample size

To calculate the minimally required number of centres participating in the trial, we used data from a previous trial [7]. Calculations were based on the normal approximation to the binomial distribution, using a type I error risk (alpha) of 5%, and 80% power. Based on the results, we aimed to include at least 19 centres that would treat 350 CR patients, on average, during the study period of 1 year. Further details can be found in [20].

### Cluster randomization and allocation

**Fig. 2:**
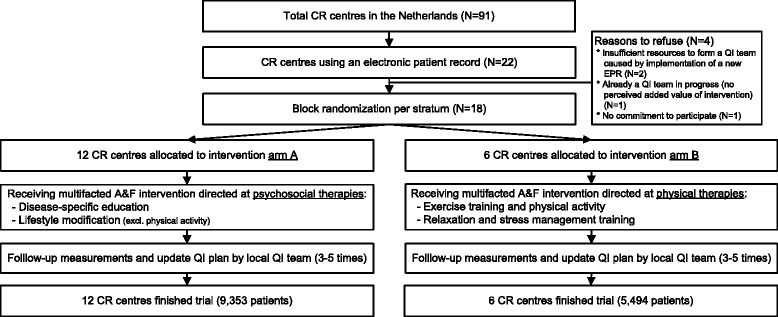
Flow of centres through the trial

Randomization of centres was stratified by size (more versus less than 30 patients starting treatment per month) (Fig. 2). Per stratum, we generated a randomization scheme with randomly assigned block sizes of either two or four centres using dedicated software. This scheme was concealed to those enrolling and allocating centres [20]. Due to the nature of the intervention, it was not possible to blind participants, or those involved in providing the intervention, to allocation.

### Statistical analysis

To assess the effect of the intervention, we performed separate mixed effects logistic regression analyses [13, 28] for each of the care processes and patient outcomes (primary outcome) and four therapies (secondary outcome) for which exactly one study group received feedback. To this end, we included covariates ‘study group’, ‘time’, and ‘study group × time’. We focused on the interaction term to assess the difference in change over 1-year study follow-up between the two groups—that is, the effect of the intervention—because we expected clinical performance to improve gradually as a result of our intervention. We used random effects to adjust for the variation in baseline performance between centres (random intercept for each centre) and the variation in effect over time (random slope for time). To adjust for differences in case mix, we included age, gender, and indication for CR at patient level and size (average weekly patient volume) and type (specialized rehabilitation centre or part of a university or teaching hospital versus part of a non-teaching hospital) at centre level as covariates.

To assess the effects on the overall performance (number of indicators at or above benchmark level) and data completeness (number of indicators for which centres recorded complete data), we used mixed effects linear regression. Per centre, we assessed for both the change in percentage between baseline and at 1-year study follow-up. Additionally, we explored secular trends in the four patient-level general processes, that were shown to both groups, by performing mixed effects logistic analyses while withholding ‘study group’ and ‘study group × time’ as covariates. Changes in the five centre-level processes were assessed by counting the number of such processes that were in place at baseline and follow-up. Finally, we performed separate mixed effects logistic regression analyses to assess in concordance with guideline recommendations for attendance of each of the four CR therapies as measured at the end of the programme.

We used Multiple Imputation by Chained Equations (MICE) to handle missing data on outcomes and confounders [29]. To verify the robustness of our findings, we performed a sensitivity analysis with complete cases only. All analyses were performed using R version 3.1.2 (R Foundation for Statistical Computing; Vienna, Austria).

## Results

### Participants

Eighteen of 22 eligible CR centres accepted our invitation to participate in the trial. Our randomization scheme assigned ten centres to group A (receiving feedback with respect to psychosocial rehabilitation) and eight to group B (receiving feedback with respect to physical rehabilitation). However, due to an algorithmic error in our software, two centres in group B received the intervention associated with group A, leading to an eventual distribution of 12 centres in group A and six in group B (see Fig. 1). Table 1 shows the baseline characteristics of centres and patients. The distribution of all characteristics, except for centre type, was equal between the groups. During the study period, a total of 14,847 patients started CR in the participating centres.

**Table 1:** Baseline characteristics of centres (*N* = 18) and patients (*N* = 14,847) per study group; values are numbers (%), unless indicated otherwise

Characteristics	Group A (feedback on psychosocial rehabilitation)	Group B (feedback on physical rehabilitation)
Centres		
Number participating	12 (66.6)	6 (33.3)
Median (min-max) number of patients per year	431 (183–1156)	370 (256–988)
Large centre (>30 patients per month)	6 (50.0)	3 (50.0)
Centre type		
Non-teaching hospital	7 (58.3)	3 (50.0)
Teaching hospital	2 (16.7)	3 (50.0)
University hospital or specialized rehabilitation centre	3 (25.0)	0 (0.0)
Patients		
Number included in analyses	9353	5494
Mean (SD) age in years	65.0 (11.5)	65.9 (11.8)
Male gender	6650 (71.1)	3900 (71.0)
Indications for CR		
ACS with revascularization	4689 (50.1)	2620 (47.7)
ACS without revascularization	469 (5.0)	401 (7.3)
Elective CABG or valvular surgery	1346 (14.4)	637 (11.6)
Elective PCI	536 (5.7)	341 (6.2)
Other elective interventions	360 (3.8)	119 (2.2)
CHF or stable AP, no intervention	262 (2.8)	194 (3.5)
Other diagnosis, no intervention	456 (4.9)	163 (3.0)
Unknown	1235 (13.2)	1019 (18.5)

*Abbreviations*: *ACS* acute coronary syndrome, *AP* angina pectoris, *CABG* coronary artery bypass graft surgery, *CHF* chronic heart failure, *PCI* percutaneous coronary intervention, *SD* standard deviation

### Implementation of the intervention

Table 2 shows detailed information on how, and to what extent, the main components of the A&F intervention were implemented in the participating centres. There were no differences between the study groups in their mean study period, size of the local multidisciplinary teams, and attendance to the educational outreach visits. Local multidisciplinary teams consisted of 7.1 members on average. isits were attended by 5.1 (74%) members on average, but we observed a decrease in attendance over time from 5.83 (84%) members in the first visit to 5.1 (72%) in the fourth. Among the attendants, there were typically a nurse, physiotherapist, cardiologist or manager, and a psychologist or social worker; sometimes, also, a dietician, sports physician, or medical secretary attended. Cardiologists and/or managers could typically only attend for 30 to 60 min. As it turned out challenging to plan visits at a time during which sufficient team members were available, the average duration for A&F iterations was 4.0 months (SD 1.4) instead of the intended 3 months. For the same reason, one centre in group A completed only three A&F iterations instead of the per protocol minimum of four iterations. There were no differences between study groups in the number of indicators that teams selected into their QI plan nor in the number of actions they planned for each of those indicators. The mean number of indicators in a QI plan decreased from 8.0 (SD 2.4) during the first A&F iteration to 5.0 (SD 3.2) in the final iteration. Teams reported to have achieved their improvement goals for 1.8 indicators per A&F iteration on average. The complete study population achieved 21.0% (49/233) of the goals they set within the study period; in group A, this was 15.6% (24/154) compared to 31.6% (25/79) in group B (*χ* ^2^ = 8.110, *df* = 1, *P* = 0.004).

**Table 2:** Implementation of the multifaceted A&F intervention, separately per study group; values are mean (SD)

Implementation of the A&F intervention	Group A (feedback on psychosocial rehabilitation)		Group B (feedback on physical rehabilitation)	
		Range		Range
Multidisciplinary teams				
Length of study period per centre in months	19.8 (6.0)	12–30	22.5 (4.1)	14–27
Number of A&F iterations	4.6 (1.0)	3–6	5.7 (0.7)	4–6
Size of local multidisciplinary team	7.5 (2.8)	3–13	6.3 (1.3)	4–8
Number of team members attending outreach visits	5.4 (1.9)	1–11	4.7 (1.8)	2–8
Number (%) of teams receiving first telephone follow-up	5 (41.7)	N.A.	5 (83.3)	N.A.
Number (%) of teams receiving second telephone follow-up	3 (25.0)	N.A.	5 (83.3)	N.A.
Quality improvement plans				
Number of goals set (number of areas for improvement included in plan)	6.9 (3.1)	1–14	6.3 (2.5)	0–10
Mean number of planned actions per goal	1.9 (0.5)	1.0–3.3	1.6 (0.4)	1.0–2.6
Number of goals achieved per A&F iteration	1.7 (1.5)	0–5	1.9 (1.5)	0–6
Number of goals unachieved at study end	5.9 (3.5)	1–13	3.5 (2.2)	0–7

*Abbreviations*: *A&F* audit and feedback, *N.A.*. not applicable, *SD* standard deviation

### Effects on clinical performance

Table 3 shows the effect on clinical performance as measured by the 11 care process and six patient outcome indicators. For none of the care processes nor patient outcomes in our study, the intervention led to significant differences in performance between study groups. We observed a positive secular trend for indicator 8 ‘Patients receive a discharge letter with remaining lifestyle goals’ in both the control (OR 5.39; 95% CI 2.14 to 13.56) and intervention group (OR 4.61; 95% CI 2.29 to 9.30). In the control group, we observed positive secular trends for indicators 12 ‘Completion of stress management and relaxation therapy’ (OR 2.47; 95% CI 1.25 to 4.88 per year), 14 ‘Improvement in exercise capacity’ (OR 1.28; 95% CI 1.11 to 1.47), and 17 ‘Vigorously active lifestyle norm met at discharge’ (OR 1.29; 95% CI 1.15 to 1.45). We found negative trends for indicator 11 ‘Exercise training completed’ in the control (OR 0.44; 95% CI 0.27 to 0.74) and for indicator 4 ‘Disease specific education completed’ in the intervention (OR 0.44; 95% CI 0.29 to 0.67) group. Our sensitivity analysis for clinical performance showed similar results (see Appendix 1); we found a positive secular trend in the control group for indicator 17 and a negative trend for indicator 4 in both the control and intervention group and no significant differences in performance between groups.

**Table 3:** Effects on clinical performance measured by 11 care processes and six patient outcomes (primary outcome) (*n* = 14,874)

Care processes and patient outcomes	Type	Control group			Intervention group			*P* value
		Crude baseline performance	Crude follow-up performance	Adjusted performance trend [OR (95% CI)]	Crude baseline performance	Crude follow-up performance	Adjusted performance trend [OR (95% CI)]	
Psychosocial rehabilitation (study group A)								
1. Complete data on psychological functioning	Process	69.0% (623/903)	68.3% (3134/4591)	0.64 (0.32 to 1.28)	86.1% (1733/2012)	84.7% (6217/7341)	0.69 (0.42 to 1.11)	0.872
2. Complete data on social functioning	Process	16.1% (145/903)	15.5% (712/4591)	0.17 (0.01 to 2.06)	53.2% (1071/2012)	56.4% (4142/7341)	1.36 (0.05 to 3.71)	0.130
3. Complete data on lifestyle factors	Process	82.8% (748/903)	84.6% (3885/4591)	0.92 (0.46 to 1.85)	86.1% (1733/2012)	80.2% (5884/7341)	1.03 (0.57 to 1.86)	0.815
4. Disease specific education completed^a^	Process	51.3% (424/827)	44.1% (1796/4071)	0.76 (0.48 to 1.20)	62.7% (842/1342)	61.7% (3046/4934)	0.44 (0.29 to 0.67)	0.091
5. Lifestyle modification programme completed^a^	Process	41.3% (373/903)	40.8% (1874/4591)	1.08 (0.63 to 1.85)	55.7% (877/1575)	58.3% (3255/5580)	1.34 (0.78 to 2.31)	0.385
6. Improved quality of live after CR	Outcome	44.6% (403/903)	46.5% (2133/4591)	0.99 (0.86 to 1.13)	41.0% (826/2012)	43.5% (3193/7341)	1 (0.89 to 1.12)	0.571
7. Successful smoking cessation	Outcome	56.0% (506/903)	54.2% (2489/4591)	0.96 (0.84 to 1.09)	49.5% (996/2012)	51.1% (3752/7341)	0.99 (0.89 to 1.11)	0.553
8. Patients receive a discharge letter with remaining lifestyle goals	Process	13.1% (118/903)	19.9% (914/4591)	5.39 (2.14 to 13.56)	27.3% (549/2012)	28.9% (2120/7341)	4.61 (2.29 to 9.30)	0.764
Physical rehabilitation (study group B)								
9. Complete data on physical functioning	Process	54.5% (1097/2012)	61.8% (4534/7341)	1.35 (0.72 to 2.54)	52.6% (475/903)	65.6% (3010/4591)	1.78 (0.75 to 4.20)	0.611
10. Complete data concerning cardiovascular risk factors	Process	55.3% (1112/2012)	49.8% (3656/7341)	1.12 (0.76 to 1.65)	54.4% (491/903)	60.4% (2775/4591)	1.34 (0.83 to 2.17)	0.562
11. Exercise training completed^a^	Process	53.4% (1074/2012)	45.9% (3366/7341)	0.44 (0.27 to 0.74)	65.8% (421/640)	60.9% (1780/2922)	0.8 (0.29 to 2.21)	0.374
12. Relaxation and stress management training completed^a^	Process	43.6% (666/1528)	48.3% (2367/4898)	2.47 (1.25 to 4.88)	44.7% (370/827)	45.8% (1863/4071)	1.04 (0.40 to 2.71)	0.183
13. Cardiovascular risk factors evaluated at discharge	Process	10.3% (208/2012)	7.4% (541/7341)	0.87 (0.41 to 1.83)	32.7% (295/903)	29.9% (1373/4591)	1.01 (0.43 to 2.37)	0.667
14. Improvement in exercise capacity	Outcome	46.5% (936/2012)	51.3% (3769/7341)	1.28 (1.11 to 1.47)	50.2% (453/903)	49.7% (2280/4591)	1.12 (0.91 to 1.38)	0.248
15. Successful work resumption	Outcome	53.3% (1072/2012)	52.4% (3848/7341)	0.93 (0.83 to 1.05)	65.7% (593/903)	68.5% (3146/4591)	0.97 (0.84 to 1.12)	0.448
16. Moderately active lifestyle norm met at discharge	Outcome	37.1% (747/2012)	35.7% (2621/7341)	0.96 (0.83 to 1.11)	71.8% (648/903)	72.8% (3340/4591)	1 (0.85 to 1.17)	0.633
17. Vigorously active lifestyle norm met at discharge	Outcome	22.1% (444/2012)	30.8% (2263/7341)	1.29 (1.15 to 1.45)	26.8% (242/903)	28.1% (1292/4591)	1.13 (0.99 to 1.30)	0.200

We used Multiple Imputation by Chained Equations to handle missing data. Baseline period: first 3 months of study period; follow-up period: complete study period minus the baseline period. Performance trends: odds ratios associated with a 1-year study follow-up, adjusted for patients’ age, gender, indication for CR, and centres’ type and size. *P* value: probability of trends being similar in intervention and control group

*Abbreviations*: *CI* confidence interval, *CR* cardiac rehabilitation, *OR* odds ratio

^a^We excluded centres with incomplete data for this indicator. The number of centres and patients included in the analyses was therefore as follows: indicator 4: 12 centres, 11,174 patients; indicator 5: 15 centres, 12,649 patients; indicator 11: 16 centres, 12,915 patients; and indicator 12: 15 centres, 11,324 patients

Overall clinical performance did not significantly improve in centres (effect 4.1% per year; 95% CI −1.13 to 8.53). Data completeness improved by 4.5% per year (95% CI 0.65 to 8.36). Appendix 2 shows the secular trends in the five general processes that were shown to both groups. We found a positive effect for indicator 21 ‘Cardiologist and GP receive a report after CR’ (OR 3.42; 95% CI 2.24 to 5.24) and a negative effect for indicators 18 ‘Median time between hospital discharge and needs assessment procedure’ (OR 0.7; 95% CI 0.54 to 0.91) and 20 ‘Rehabilitation evaluated at discharge’ (OR: 0.43; 95% CI 0.28 to 0.64). In the complete case analysis (Appendix 4), we did not find any significant effect.

Table 4 shows the effects on guideline concordance with respect to each of the four therapies. In the control group, we observed a positive concordance trend for prescribing exercise therapy (OR 2.52; 95% CI 1.03 to 6.16). We found negative trends for prescribing disease-specific education (OR 0.62; 95% CI 0.43 to 0.89) and lifestyle modification (OR 0.37; 95% CI 0.15 to 0.92) in the intervention group. Concerning concordance with respect to therapy attendance, we found a negative trend in the control group for relaxation and stress management (OR 0.44; 95% CI 0.23 to 0.83) and in the intervention group for disease-specific education (OR 0.51; 95% CI 0.32 to 0.81). For none of the therapies, the intervention led to significant differences in concordance trends, for neither prescription nor attendance. Overall, concordance rates for prescription of all four therapies were higher compared to attendance rates. Concordance rates were highest for prescribing relaxation and stress management (85.1%) followed by education (77.8%). The lifestyle modification showed the lowest concordance rates, for both prescription (44.4%) and attendance (37.4%). Our sensitivity analysis for guideline concordance showed a concordance improvement for attendance of education (OR 2.83; 95% CI 1.10 to 7.27) and a negative concordance trend in the control group for attendance of both the lifestyle modification (OR 0.72; 95% CI 0.53 to 0.97) and relaxation and stress management therapy (OR 0.42; 95% CI 0.17 to 0.99) (Appendix 3).

**Table 4:** Concordance rates and difference in concordance between study groups for the four CR therapies (both prescribed and attended therapies) (secondary outcome) (*n* = 14,874)

CR therapies	Control group			Intervention group			*P* value
	Crude baseline concordance	Crude follow-up concordance	Adjusted concordance trend [OR (95% CI)]	Crude baseline concordance	Crude follow-up concordance	Adjusted concordance trend [OR (95% CI)]	
Psychosocial rehabilitation (study group A)							
Education (prescribed)	73.4% (663/903)	66.0% (3031/4591)	0.66 (0.43 to 1.01)	85.8% (1727/2012)	85.8% (6300/7341)	0.62 (0.43 to 0.89)	0.830
Lifestyle (prescribed)	28.9% (261/903)	24.9% (1145/4591)	0.49 (0.14 to 1.80)	62.5% (1258/2012)	61.2% (4489/7341)	0.37 (0.15 to 0.92)	0.716
Education (attended)^a^	74.6% (617/827)	68.3% (2780/4071)	0.98 (0.55 to 1.74)	53.7% (720/1342)	52.0% (2566/4934)	0.51 (0.32 to 0.81)	0.099
Lifestyle (attended)^a^	26.3% (168/640)	28.2% (824/2922)	1.19 (0.74 to 1.91)	43.5% (876/2012)	51.5% (3777/7341)	0.83 (0.64 to 1.08)	0.187
Physical rehabilitation (study group B)							
Exercise (prescribed)	72.1% (1450/2012)	80.0% (5869/7341)	2.52 (1.03 to 6.16)	41.2% (372/903)	49.4% (2269/4591)	0.85 (0.24 to 3.07)	0.176
Relaxation (prescribed)	88.9% (1789/2012)	92.8% (6811/7341)	0.91 (0.70 to 1.17)	78.5% (709/903)	80.1% (3679/4591)	1.03 (0.79 to 1.35)	0.447
Exercise (attended)^a^	61.2% (935/1528)	61.3% (3003/4898)	0.82 (0.61 to 1.09)	46.8% (387/827)	49.7% (2022/4071)	0.92 (0.60 to 1.39)	0.565
Relaxation (attended)^a^	57.0% (898/1575)	48.3% (2695/5580)	0.44 (0.23 to 0.83)	75.4% (681/903)	74.4% (3416/4591)	0.97 (0.47 to 2.01)	0.147

We used Multiple Imputation by Chained Equations to handle missing data. Baseline period: first 3 months of study period; follow-up period: complete study period minus the baseline period. Performance trends: odds ratios associated with a 1-year study follow-up, adjusted for patients’ age, gender, indication for CR, and centres’ type and size. *P* value: probability of trends being similar in intervention and control group

*Abbreviations*: *CI* confidence interval, *CR* cardiac rehabilitation, *OR* odds ratio

^a^We excluded centres with incomplete data for this therapy. The number of centres and patients included in the analyses was therefore as follows: education: 12 centres, 11,174 patients; lifestyle modification: 15 centres, 12,649 patients; exercise training: 16 centres, 12,915 patients; and relaxation and stress management training: 15 centres, 11,324 patients

## Discussion

We evaluated an A&F intervention in a large cluster-randomized trial among 18 CR centres and 14,847 patients. Our intervention modestly improved data completeness and engaged teams to set improvement goals, but it yielded no improvement of clinical performance by multidisciplinary CR teams.

A Cochrane review of 140 randomized A&F trials showed a median effect of 4.3% improvement in quality of care, with a minority of studies showing a strong positive effect [15]. In the review, the authors identified characteristics that may enhance A&F effectiveness, such as the use of educational outreach visits, providing feedback multiple times, and involving the entire team in action planning and goal setting activities [15–19]. We incorporated all of these characteristics in our intervention. Additionally, we built on the findings of an extensive barrier analysis which identified the need to target decision-making by multidisciplinary teams in order to increase guideline concordance in the field of CR [14]. The resulting intervention encouraged multidisciplinary teams to develop and revise (up to five times) improvement plans based on indicator-based performance that was provided in quarterly feedback reports. Less than 20% of similar studies use iterative cycles of change, and only 14% of them repeatedly use data over time [30]. However, despite our efforts to design an effective intervention, the intervention did not improve clinical performance. Apparently, there are other, unidentified factors that are equally or more important to achieve change in clinical practice.

The authors of the Cochrane review recommended that the development and evaluation of A&F interventions should be informed by the explicit use of theory [15]. Although we designed our study before this recommendation was published, our intervention is well-founded in the Model for Improvement [25]. The model encourages teams improving their practice following plan-do-study-act (PDSA) cycles. This also fits within Control Theory [31], which poses that A&F effects are achieved through a mechanism of three steps: (i) performance feedback convinces health professionals that change is necessary and to set improvement intentions, (ii) intentions are translated into action, and (iii) action impacts the outcome of interest. We contributed to a more in-depth understanding of Control Theory by performing a quantitative process evaluation alongside our trial [32]. While performance scores and benchmark comparisons clearly influenced health professionals’ improvement intentions, a substantial amount of feedback information was lost in the translation to improvement intentions because professionals disagreed with the benchmarks, deemed improvement unfeasible, or did not consider the indicator an essential aspect of care quality [32]. This consequently impeded intentions to improve practice, i.e. the first step in the mechanism posed by Control Theory, and thus explains part of the ineffectiveness of our intervention. The current study further revealed that another part of the ineffectiveness can be explained by the fact that professionals were not able to translate their intentions into completed actions, i.e. the second step of the mechanism, before the study end.

The electronic nature of our intervention enabled us to monitor and measure improvement processes at the centre level: teams selected areas for improvement and planned and managed their QI activities within the same web-based system through which they were provided with performance feedback. By doing so, teams were able to develop a QI plan entirely tailored to their local organization. Nevertheless, we found that our intervention successfully encouraged teams to define local performance improvement goals, but it largely failed to support them with actually completing the actions needed to achieve those goals: 79% of intended actions remained uncompleted until the end of the study. Previous research in the field of intensive care [33, 34] and general practice [35, 36] suggested that failures to complete improvement actions may be due to organizational barriers such as competing priorities or a lack of leadership or professional barriers such as a lack of individual skills or knowledge to take effective improvement actions. Our A&F intervention did not completely solve these barriers. The professional barriers may be reduced by extending the intervention with ready-to-use improvement tools [16, 33]. The few A&F studies that incorporated such support did so in different ways: through facilitated group discussions to reflect upon the feedback and identify improvement strategies [37] and by including suggestions in the feedback reports for how to address deficiencies in practice [38]. As the surplus value of adding supportive improvement tools to A&F interventions has not yet been investigated, we suggest that this be a focus of future research. Examples of persisting organizational barriers within our study context were related to lacks of resources (e.g. budget ceilings imposed by insurers), competing interests between managers from different clinical disciplines, and poor attendance of clinical leadership (cardiologists and managers) at outreach visits. This was supported by a qualitative study in the context of our intervention, which revealed that participants considered team commitment and organizational readiness important yet difficult factors to operationalize [39]. Recently, socio-technical frameworks have been proposed to design and evaluate A&F interventions, such as the Triangle Model [40], Team Strategies and Tools to Enhance Performance and Patient Safety (TeamSTEPPS) framework [41], the Systems Engineering Initiative for Patient Safety (SEIPS) model [42] and the 8-dimension socio-technical model [43]. Such socio-technical models typically approach the implementation process as consisting of multiple components that continuously interact with and change each other, including people, teams, tasks, tools and technologies, underlying organizational conditions, and the surrounding context. To address this complexity, future studies could consider using socio-technical models as the underlying theoretical framework to guide the development, implementation, and evaluation of A&F interventions.

A limitation of our study is that we implemented our A&F intervention shortly after centres had started working with a new EPR. Although the reuse of routinely collected EPR data has minimized data collection burden for participating clinicians, the EPR implementation may have conflicted with the time and resources available for working on actual performance improvement. Second, some outcome measures showed little room for improvement (i.e. ceiling effects), making them less likely to change significantly over the course of the study, and as such less suitable for assessing the effectiveness of our intervention. Other outcome measures might have been difficult to improve because they relied on patients’ compliance with prescribed therapies, which is a well-known barrier to guideline concordance [44]. Third, there may have contamination between groups due to an overall increase of awareness of clinical performance and quality improvement. This may have resulted in professionals working on other aspects of CR care, even though they had been randomized to target only psychosocial (group A) or physical rehabilitation (group B). Fourth, we included one centre less in our study sample than estimated in our sample calculations. Although we exceeded the estimated required number of patients per centre, we cannot rule out lack of statistical power as a potential explanation for finding no significant effects. Finally, two centres were incorrectly assigned to group A due to an algorithmic error in our software. However, since there were no differences in baseline characteristics between study groups, we believe the unequal distribution of centres did not influence our final results.

## Conclusions

We designed a web-based A&F intervention in the field of CR guided by an extensive analysis of barriers in the field and by incorporating characteristics proven successful in the A&F literature. The intervention had no effect on the measured care processes, patient outcomes, or guideline concordance. Our intervention did modestly increase data completeness and engaged teams to define local performance improvement goals but failed to support them in actually completing the improvement actions that were needed to achieve those goals. Future studies should focus on improving A&F interventions and their evaluation, for instance by improving the actionability of feedback on clinical performance and by addressing the socio-technical perspective of implementation processes more extensively.

## Appendix 1

**Table 5:** Effect on clinical performance measured by 11 care processes and six patient outcomes (primary outcome): complete case analysis including correction for case mix

Care processes and patient outcomes	Type	Control group			Intervention group			*P* value	*N* (centres)	Missing values
		Crude baseline performance	Crude follow-up performance	Adjusted performance trend [OR (95% CI)]	Crude baseline performance	Crude follow-up performance	Adjusted performance trend [OR (95% CI)]			
Psychosocial rehabilitation (study group A)										
1. Complete data on psychological functioning	Process	67.1% (425/633)	70.4% (2703/3842)	0.56 (0.25 to 1.25)	86.8% (1474/1698)	85.6% (5495/6420)	0.82 (0.31 to 2.17)	0.690	12,593 (18)	0 (0%)
2. Complete data on social functioning	Process	20.2% (128/633)	17.3% (666/3842)	0.14 (0.01 to 1.91)	56.2% (955/1698)	56.2% (3608/6420)	0.12 (0.01 to 1.95)	0.131	12,593 (18)	0 (0%)
3. Complete data on lifestyle factors	Process	83.3% (527/633)	86.0% (3304/3842)	0.90 (0.45 to 1.83)	86.3% (1465/1698)	80.7% (5180/6420)	0.79 (0.32 to 1.98)	0.619	12,593 (18)	0 (0%)
4. Disease specific education completed^a^	Process	36.3% (61/168)	46.5% (451/970)	0.59 (0.44 to 0.77)	48.9% (149/305)	69.4% (738/1064)	0.39 (0.26 to 0.60)	0	2507 (10)	1589 (38.8%)
5. Lifestyle modification programme completed^a^	Process	51.4% (19/37)	42.7% (108/253)	0.63 (0.17 to 2.42)	21.3% (61/286)	33.3% (235/706)	1.24 (0.19 to 8.01)	0.819	1282 (9)	1541 (54.6%)
6. Improved quality of live after CR	Outcome	47.0% (70/149)	48.8% (471/966)	1 (0.76 to 1.33)	46.6% (275/590)	49.0% (1182/2412)	0.96 (0.69 to 1.33)	0.793	4117 (17)	8476 (67.3%)
7. Successful smoking cessation	Outcome	63.0% (63/100)	58.3% (277/475)	0.91 (0.65 to 1.29)	54.1% (119/220)	58.5% (424/725)	0.84 (0.56 to 1.26)	0.392	1520 (17)	11,073 (87.9%)
8. Patients receive a discharge letter with remaining lifestyle goals	Process	9.1% (24/263)	17.8% (209/1173)	5.35 (0.58 to 49.38)	15.5% (161/1037)	26.7% (1004/3755)	2.06 (0.13 to 31.63)	0.609	6228 (18)	6365 (50.5%)
Physical rehabilitation (study group B)										
9. Complete data on physical functioning	Process	56.0% (950/1698)	64.1% (4118/6420)	1.66 (0.90 to 3.05)	57.8% (366/633)	67.8% (2605/3842)	0.97 (0.35 to 2.73)	0.956	12,593 (18)	0 (0%)
10. Complete data concerning cardiovascular risk factors	Process	53.5% (909/1698)	48.6% (3123/6420)	1.13 (0.76 to 1.70)	54.8% (347/633)	62.9% (2415/3842)	0.86 (0.45 to 1.65)	0.657	12,593 (18)	0 (0%)
11. Exercise training completed^a^	Process	55.0% (364/662)	50.2% (1124/2239)	1.17 (0.49 to 2.77)	75.0% (3/4)	NA (0/0)	0 (0 to Inf)	1	2905 (9)	2929 (50.2%)
12. Relaxation and stress management training completed^a^	Process	35.0% (143/409)	51.4% (721/1404)	0.40 (0.09 to 1.72)	40.0% (6/15)	32.7% (145/443)	3.56 (0.16 to 79.38)	0.418	2271 (11)	518 (18.6%)
13. Cardiovascular risk factors evaluated at discharge	Process	12.1% (156/1286)	8.8% (360/4085)	0.51 (0.10 to 2.66)	48.7% (218/448)	41.1% (970/2359)	0.49 (0.04 to 5.46)	0.567	8178 (18)	4415 (35.1%)
14. Improvement in exercise capacity	Outcome	50.8% (199/392)	59.5% (966/1624)	1.14 (0.95 to 1.36)	57.1% (96/168)	51.0% (490/960)	1.22 (0.89 to 1.68)	0.224	3144 (12)	9449 (75.0%)
15. Successful work resumption	Outcome	65.2% (204/313)	59.8% (539/901)	1.07 (0.80 to 1.44)	80.4% (86/107)	81.1% (456/562)	0.86 (0.51 to 1.46)	0.577	1883 (17)	10,710 (85.1%)
16. Moderately active lifestyle norm met at discharge	Outcome	27.3% (229/839)	32.0% (781/2439)	1.45 (0.98 to 2.13)	75.9% (271/357)	75.7% (1249/1650)	1.26 (0.69 to 2.33)	0.457	5285 (17)	7308 (58.0%)
17. Vigorously active lifestyle norm met at discharge	Outcome	9.2% (77/834)	19.2% (467/2438)	1.36 (1.04 to 1.79)	21.7% (74/341)	21.8% (354/1623)	1.32 (0.92 to 1.90)	0.129	5236 (17)	7357 (58.4%)

Baseline period: first 3 months of study period; follow-up period: complete study period minus the baseline period. Performance trends: odds ratios associated with a 1-year study follow-up, adjusted for patients’ age, gender, indication for CR, and centres’ type and size. *P* value: probability of trends being similar in intervention and control group

*Abbreviations*: *CI* confidence interval, *CR* cardiac rehabilitation, *OR* odds ratio, *NA* not applicable

We excluded centres with incomplete data for this indicator. The number of centres and patients included in the analyses was therefore as follows: indicator 4: 12 centres, 11,174 patients; indicator 5: 15 centres, 12,649 patients; indicator 11: 16 centres, 12,915 patients; and indicator 12: 15 centres, 11,324 patients

## Appendix 2

**Table 6:** Results and secular trend on five general CR processes all centres received feedback upon: using MICE including correction for case mix and results on availability of five organizational structures

Care processes and organizational structures	Type	Crude baseline performance	Crude follow-up performance	Performance trend [OR (95% CI)]	*N* (centres)
Indicators referring to general practices (both study groups)					
18. Median time between hospital discharge and needs assessment procedure	Process	68.0% (1983/2915)	61.6% (7355/11,932)	0.7 (0.54 to 0.91)	14,847 (18)
19. Patients are offered a rehabilitation plan tailored to their needs	Process	85.0% (2479/2915)	85.4% (10,191/11,932)	0.89 (0.72 to 1.09)	14,847 (18)
20. Rehabilitation evaluated at discharge	Process	39.0% (1137/2915)	34.0% (4059/11,932)	0.43 (0.28 to 0.64)	14,847 (18)
21. Cardiologist and GP receive a report after CR	Process	42.5% (1239/2915)	57.7% (6879/11,932)	3.42 (2.24 to 5.24)	14,847 (18)
Availability of five organizational structures (both study groups)					
22. Professionals work with a multidisciplinary patient record	Yes/no	94.4% (17/18)	100% (18/18)	N.A.	18
23. Availability of specialized education for patients with chronic heart failure	Yes/no	55.6% (10/18)	61.1% (11/18)	N.A.	18
24. Assessment of long-term patients outcomes	Yes/no	33.3% (6/18)	33.3% (6/18)	N.A.	18
25. Performing internal evaluation and quality improvement	Yes/no	83.3% (15/18)	94.4% (17/18)	N.A.	18
26. Performing patient satisfactory research	Yes/no	44.4% (8/18)	55.6% (10/18)	N.A.	18

We used Multiple Imputation by Chained Equations to handle missing data. Baseline period: first 3 months of study period; follow-up period: complete study period minus the baseline period. Performance trends: odds ratios associated with a 1-year study follow-up, adjusted for patients’ age, gender, indication for CR, and centres’ type and size

*Abbreviations*: *CI* confidence interval, *CR* cardiac rehabilitation, *GP* general practitioner, *OR* odds ratio, *NA*. not applicable

## Appendix 3

**Table 7:** Concordance rates and difference in concordance between study groups for the four CR therapies (both prescribed and attended therapies) (secondary outcome): complete case analysis including correction for case mix

CR therapies	Control group			Intervention group			*P* value	*N* (centres)	Missing ( %)
	Crude baseline concordance	Crude follow-up concordance	Adjusted concordance trend [OR (95% CI)]	Crude baseline concordance	Crude follow-up concordance	Adjusted concordance trend [OR (95% CI)]			
Psychosocial rehabilitation (study group A)									
Education (prescribed)	81.2% (375/462)	71.1% (2292/3222)	0.69 (0.38 to 1.24)	87.5% (1411/1612)	89.9% (5347/5951)	0.84 (0.39 to 1.83)	0.662	11,247 (18)	1346 (10.7%)
Lifestyle (prescribed)	34.3% (155/452)	25.6% (830/3240)	0.45 (0.1 to 1.95)	63.0% (1023/1624)	61.8% (3693/5978)	1.45 (0.24 to 8.65)	0.682	11,294 (18)	1299 (10.3%)
Education (attended)	83.8% (150/179)	77.8% (782/1005)	1.19 (0.58 to 2.45)	62.1% (185/298)	79.1% (828/1047)	2.83 (1.1 to 7.27)	0.031	4804 (11)	1567 (38.3%)
Lifestyle (attended)	21.8% (27/124)	21.8% (157/722)	0.72 (0.53 to 0.97)	28.1% (125/445)	32.7% (384/1174)	1.25 (0.86 to 1.84)	0.243	3834 (9)	3369 (57.8%)
Physical rehabilitation (study group B)									
Exercise (prescribed)	72.6% (976/1345)	82.8% (4211/5088)	2.66 (0.86 to 8.26)	38.8% (124/320)	51.9% (1271/2451)	2.81 (0.38 to 20.67)	0.310	9204 (18)	3389 (26.9%)
Relaxation (prescribed)	89.9% (1460/1625)	95.0% (5724/6026)	1.49 (0.9 to 2.47)	82.6% (399/483)	83.8% (2805/3348)	1.4 (0.66 to 2.97)	0.378	11,482 (18)	1111 (8.8%)
Exercise (attended)	75.2% (279/371)	64.1% (667/1040)	0.69 (0.43 to 1.1)	75.0% (12/16)	61.0% (269/441)	0.64 (0.26 to 1.57)	0.326	3976 (14)	921 (33.0%)
Relaxation (attended)	76.7% (227/296)	81.2% (574/707)	0.42 (0.17 to 0.99)	100% (46/46)	94.1% (272/289)	0.41 (0.1 to 1.61)	0.203	5514 (12)	1485 (52.6%)

Baseline period: first 3 months of study period; follow-up period: complete study period minus the baseline period. Performance trends: odds ratios associated with a 1-year increase in the exposure, adjusted for patients’ age, gender, indication for CR, and centres’ type and size. *P* value: probability of trends being similar in intervention and control group

*Abbreviations*: *CI* confidence interval, *CR* cardiac rehabilitation, *OR* odds ratio

## Appendix 4

**Table 8:** Results and secular trend on five general CR processes all centres received feedback upon (additional analysis): complete case analysis including correction for case mix

Care processes	Type	Crude baseline performance	Crude follow-up performance	Performance trend [OR (95% CI)]	*N* (centres)	Missing values
Indicators referring to general processes (both study groups)						
18. Median time between hospital discharge and needs assessment procedure	Process	64.0% (1137/1778)	60.3% (5212/8646)	0.77 (0.57 to 1.05)	10,424 (18)	2169 (17.2%)
19. Patients who are offered a rehabilitation plan tailored to their needs	Process	87.3% (2034/2331)	88.4% (9073/10,262)	0.92 (0.74 to 1.13)	12,593 (18)	0 (0%)
20. Patients who had their rehabilitation goals evaluated after CR	Process	41.1% (957/2331)	34.7% (3565/10,262)	1.25 (0.90 to 1.74)	8178 (18)	0 (0%)
21. Patients for whom their cardiologist and GP receive a report after CR	Process	38.4% (444/1156)	51.2% (1978/3861)	1.63 (0.89 to 2.98)	5017 (18)	7576 (60.2%)

Baseline period: first 3 months of study period; follow-up period: complete study period minus the baseline period. Performance trends: odds ratios associated with a 1-year study follow-up, adjusted for patients’ age, gender, indication for CR, and centres’ type and size

*Abbreviations*: *CI* confidence interval, *CR* cardiac rehabilitation, *GP* general practitioner, *OR* odds ratio
